# Synthetic MRI in children with tuberous sclerosis complex

**DOI:** 10.1186/s13244-022-01219-2

**Published:** 2022-07-07

**Authors:** Gokcen Coban, Ekim Gumeler, Safak Parlak, Bahadir Konuskan, Jale Karakaya, Dilek Yalnizoglu, Banu Anlar, Kader K. Oguz

**Affiliations:** 1 Department of Radiology, School of Medicine, Hacettepe University, Ankara, Turkey; 2 Department of Pediatric Neurology, School of Medicine, Hacettepe University, Ankara, Turkey; 3 Department of Biostatistics, School of Medicine, Hacettepe University, Ankara, Turkey

**Keywords:** Synthetic MRI, Tubers, Subcortical radial bands, Subependymal nodules, Tuberous sclerosis

## Abstract

**Objective:**

The generation of numerous sequences and quantitative data in a short scanning time is the most potential advantage of Synthetic MRI (SyMRI). We aimed to test detection of the tubers and to determine underlying tissue characteristics, and morphometric alterations in the brain of pediatric tuberous sclerosis complex (TSC) patients, using SyMRI.

**Methods:**

Conventional brain MRI (cMRI) and SyMRI were prospectively obtained from 10 TSC patients and 18 healthy control subjects (HCs). Two neuroradiologists independently evaluated tubers on both scans. Additionally, automatically segmented volume calculation and myelin quantification, including the subcortical part of the tubers and normal-appearing brain parenchyma (NABP) of patients, were carried out using SyMRI.

**Results:**

The cMRI and SyMRI comparison showed a very good correlation on the detection of the tubers (k = 0.82–0.94). Automatic segmentation of Non-gray matter/white matter/cerebrospinal fluid (Non), %Non/brain parenchymal volume, and %Non/intracranial volume was significantly higher; however, %Myelin/intracranial volume and %Myelin/brain parenchymal volume were significantly lower in the TSC patients (*p* < 0.05). The proton density values were significantly increased, and myelin fraction volume and myelin-correlated compound values were significantly decreased in the NABP in TSC patients on myelin maps (*p* < 0.05). The white-matter volume, myelin and white-matter fractional volume, longitudinal relaxation rate, transverse relaxation rate, and myelin-correlated compound values were significantly decreased in the subcortical part of tubers on quantification maps (*p* < 0.001) in TSC patients.

**Conclusion:**

SyMRI enables the detection of cortical tubers and is a developing tool in the quantification of morphometric and tissue alterations in pediatric TSC patients with a rational scanning time.

**Supplementary Information:**

The online version contains supplementary material available at 10.1186/s13244-022-01219-2.

## Key points

- Synthetic MRI (SyMRI) can provide quantitative information of the brain and may help to determine morphometric changes in tissue components.
- SyMRI can detect the myelin amount of the brain in comparison with the conventional sequences.
- SyMRI can show the abnormality in the normal-appearing white matter of the brain.

## Background

Tuberous sclerosis complex (TSC) is a neurocutaneous syndrome occurring in 1/6000–10,000 newborns [1]. It is characterized by benign hamartomas in multiple organs including the brain. Hamartomas are composed of non-malignant and disorganized cells that usually demonstrate abnormal differentiation [2]. Lesions of TSC result from mutation of the TSC1 or TSC2 genes. These genes are negative regulators of the mammalian target of the rapamycin complex (mTORC) pathway. Abnormal mTORC activation induces the differentiation and migration of the cells in the subependymal region, the cortex, and along the cell migration pathways [3].

Magnetic resonance imaging (MRI) is the first choice to detect classical parenchymal abnormalities (cortical/subcortical tubers, subependymal nodules, subcortical radial bands, and subependymal giant cell astrocytomas (SEGA)) in TSC patients [4]. Brain MRI is required every 1–3 years according to the recommendations of the 2012 International TSC Consensus Conference [5]. However, approximately half of TSC patients present with global intellectual impairment and developmental psychopathologies [6], which make them more uncooperative in the setting of the long acquisition times.

The SyMRI sequence is named ‘quantification of relaxation times and proton density by the multi-echo acquisition of a saturation recovery using turbo spin-echo readout’ (QRAPMASTER) [7] and facilitates segmentation of the brain automatically in addition to quantitative analysis of myelin with good accuracy and reproducibility. It takes about 6 min with full head coverage. The diagnostic ability of this FDA-approved sequence was previously searched for several diseases [8–13].

We aimed to detect cortical/subcortical tubers and determine tissue alterations, including myelin, in the brains of TSC patients using SyMRI, in this study. We hypothesized that SyMRI can detect cortical/subcortical tubers comparable to conventional MRI sequences and assess morphological and tissue changes quantitatively in pediatric TSC patients.

## Materials and methods

This prospective study was approved by the Institutional Review Board (#KA-180135). Informed consent was obtained from the parents of the children.

### Participants and image acquisition

Pediatric patients with TSC, who were diagnosed and followed up by the Pediatric Neurology Department of Hacettepe University, a reference center for neurophakomatoses—between August 2019 and March 2020, enrolled in this study. Exclusion criteria were: (i) the presence of hydrocephalus, (ii) presence of any structural lesion incompatible with TSC, (iii) poor image quality, (iv) incidentally tumor in both groups, (v) signal abnormalities of WM in the healthy controls (HCs).

Ten children with TSC and 18 healthy children without neurological and systemic abnormalities participated. None of the 28 subjects received sedation or contrast media. Imaging was performed with a 1.5 T MR scanner (Aera; Siemens Healthcare, Erlangen, Germany) equipped with a 20 channel phased array head coil. Conventional MRI (cMRI) sequences included axial FLAIR (TE/TR/TI: 78/7000/2220 ms, FOV: 230 × 185 mm; slice thickness (ST)/gaps: 5/2 mm; acquisition time (AT): 4 min 6 s; and voxel size: 0.4 × 0.4 × 5 mm), axial 3D T1W MPRAGE (TE/TR/:3/1680 ms, FOV: 240 × 195 mm; ST/gaps: 1.5/0.75 mm gaps; acquisition time (AT): 5 min 8 s; and voxel size: 0.8 × 0.8 × 1.5 mm), axial T2W turbo SE (TE/TR/:199/3240 ms, FOV: 230 × 185 mm; ST/gaps:5/2 mm; AT: 3 min 42 s; and voxel size: 0.4 × 0.4 × 5 mm).

The SyMRI (QRAPMASTER) was acquired in the axial plane as follows: FOV, 230 × 183 mm; voxel size, 1.5 × 1.5 mm; TE, 14, 28, 42, 56, 70 ms; TR, 4.244 s; TI, 0.0974, 0.5846, 1.8511, 4.0919 s; saturation flip angle, 120°; and AT, 6:05 min, slice thickness/gap: 4 mm/1 mm covering full brain in 30 slices.

The cMRI was performed before SyMRI. Total scanning time was 12:56 min and 6.05 min for cMRI and SyMRI, respectively.

### Image analysis

SyMRI Diagnostic Software Version 11.2 (Synthetic MR AB, Linköping, Sweden) was used for image analysis. With this software, synthetic images are created and intracranial volume (ICV), brain parenchymal volume (BPV), brain parenchymal fraction (BPF = BPV/ICV), gray matter (GM), white matter (WM), cerebrospinal fluid (CSF), Non-GM/WM/CSF, myelin (MY), and myelin fraction (MYF = MY/BPV) are quantified [14]. Also, SyMRI enables the quantification of the proton density (PD), the longitudinal relaxation rate (R1), and the transverse relaxation rate (R2). The acquisition voxel of MRI is formed by four partial volume compartments (free water partial volume, myelin partial volume, cellular partial volume, and excess parenchymal water partial volume). Each partial volume compartment has its relaxation properties (R1, R2, and PD values). Myelin volume can be achieved, by using this method, based on the assumption of the relaxation properties of the four partial volumes in a voxel. The total volume of WM, GM, CSF, Non-GM/WM/CSF, and myelin can be calculated by multiplying the volume fractions for each tissue type. The Non-GM/WM/CSF stands for the tissue not classified as GM, WM, or CSF and contains meninges, flow voids in larger blood vessels, and lesions [15].

### Qualitative evaluation

Two blinded neuroradiologists (G.C. and E.G.) independently evaluated the presence and location of tubers on FLAIR images on both cMRI and SyMRI with a 3-week gap. For the quantitative analysis, the largest subcortical part of the tuber was chosen in consensus. Also, to search for any relationship between tuber load and brain morphometry, patients were grouped into 3 according to the number of tubers: group 1 (< 10), group 2 (from 11 to 19), group 3 (> 20).

### SyMRI evaluation

An imaging workstation (syngo. via, Siemens, Germany) was used for raw image dataset piling-up. Automatic whole-brain segmentation yielded volumes of MY, BPV, BPF, ICV, MYF, GM, and Non-GM/WM/CSF in all subjects. The colored myelin-correlated compound (MyC), GM, and WM quantification maps were displayed. The segmented tissue properties were overlaid on the synthetic (Sy) T2W sequence. Regions of interest (ROIs) were drawn on an image set and then copied to the contralateral side with the ‘mirror copy’ option available in the SyMRI Diagnostic Software Version 11.2.

First, the ROIs ranging from 0.2 ml – 0.6 ml were drawn from the right (R) and left (L) globus pallidum (GP), caudate nucleus (CN), internal capsule (IC), putamen, thalamus, and also from the center of the pons, and midbrain on the colored myelin maps overlaid on T2WI of SyMRI in patients and HCs (Additional file 1: Fig. S1). T2-lesion-free regions of each centrum semiovale (CS) were selected as ROIs of NABP.

A similar ROI (0.2 ml) was drawn on the subcortical part of the selected tubers and contralateral subcortical NABP on myelin quantification maps overlaid on the FLAIR sequence from the SyMRI technique for quantitative comparison of these structures. The same measurements were repeated for the colored WM and GM quantification maps. Measurements were excluded if the contralateral homologous structure was not free of the lesion (i.e. not NABP). The MyC, WMvol, GMvol, PD, R2, and R1 within ROIs were obtained. Then, those ROIs also yielded MYFvol, WMFvol, and GMFvol, which represented the mean amount of MY, WM, and GM within a single voxel, respectively.

### Statistical analysis

Categorical variables (age, sex) were compared between groups with the Chi-square test. Quantitative variables were tested for normal distribution with the Kolmogorov–Smirnov test. Descriptive statistics were expressed as mean ± standard deviation for variables with normal distribution and median (min–max) for variables without normal distribution. For comparison, independent samples’ t test and the Mann–Whitney U test were used with normally and non-normally distributed variables, respectively. For comparison of continuous variables, the adjusted* p*-value was obtained by using the Benjamini–Hochberg (*FDR*) method [16].

The weighted kappa (K) analysis was used to evaluate interobserver agreement for MRI features. The kappa value was interpreted as poor (< 0.20), fair (0.21–0.40), moderate (0.41–0.60), good (0.61–0.80), or very good (0.81–1.00) for agreement. Confidence intervals (95% CI) of the kappa values were used to find possible non-overlapping intervals.

Comparison of the quantification (MYFvol, MyC, GMFvol, GMvol, WMFvol, WMvol, and PD, R2, R1 values) of subcortical part of the tubers and NABP was made by Wilcoxon test. The relationship of continuous and ordinal variables was examined with the Spearman rho correlation coefficient.

Among categorical variables, groups were compared using the Chi-square test. Spearman's correlation coefficient was used to determine the relationship of continuous and ordinal variables with each other.

All analyses were performed using IBM SPSS Statistics 23.0 software and R 3.6.3 program.* p*-values < 0.05 were considered statistically significant.

## Results

The TSC patients (females/males: 6/4, mean age: 11.8 ± 3.1 (range 7–17) years) and HCs (females/males: 12/6, mean age: 11 ± 3 (range 5–16) years) were similar in terms of sex (*p* = 0.683) and age (*p* = 0.507). The demographic features of the TSC patients are given in Additional file 1: Table S1.

### Qualitative evaluation

**Fig. 1:**
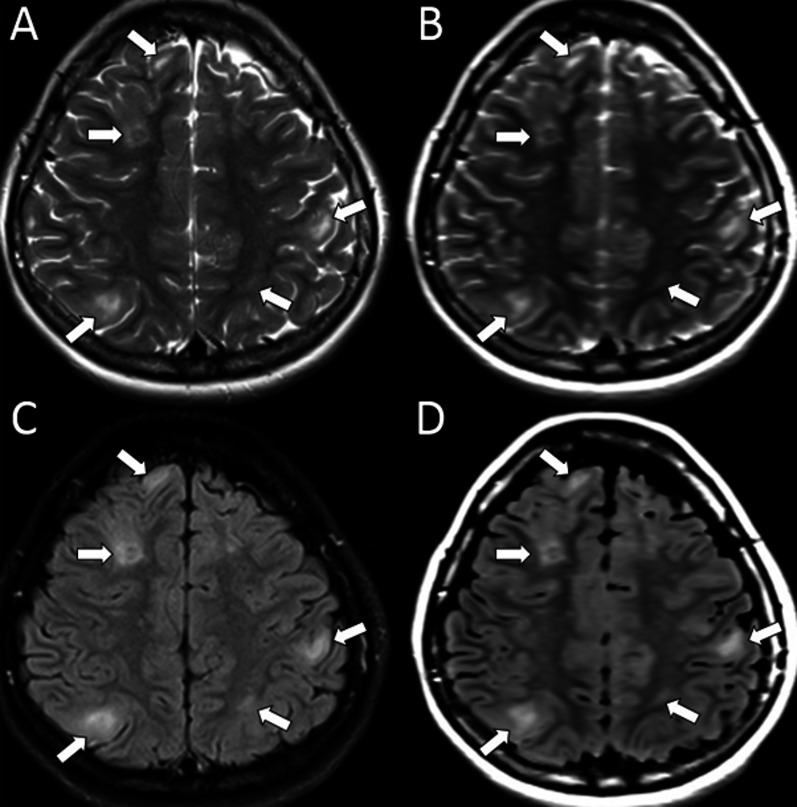
Synthetic (Sy) T2-weighted (**B**) and Sy-FLAIR (**D**) images show cortical/subcortical tubers accurately as on the conventional T2W (**A**) and FLAIR (**C**) images

Interobserver agreement for the detection of tubers on SyMRI is given in Table 1. Raters’ detection of tubers on SyMRI showed a very good correlation (k = 0.80 to 0.94), especially in the frontal lobes (Fig. 1).

**Table 1:** Conventional MRI (cMRI) versus SyMRI for cortical/subcortical tuber detection

Localization of tubers	CMRI versus SyMRI (Ob1)	CMRI versus SyMRI (Ob2)	Interobserver agreement on SyMRI	Interobserver agreement on cMRI
Frontal	0.94	0.88	0.86	0.94
Parietal	0.92	0.86	0.84	0.88
Temporal	0.88	0.80	0.80	0.84
Occipital	0.90	0.82	0.84	0.88

Ob: observer, vs: versus

The tubers in TSC patients were located in the frontal (n = 114), parietal (n = 52), temporal (n = 41), and occipital (n = 24) lobes on cMRI (Additional file 1: Table S1). By categorization based on the number of tubers, there were 2 patients in group 1, 3 patients in group 2, 5 patients in group 3. There was no significant correlation between the volumes of WM, GM, CSF, Myelin, Non-WM/GM/CSF, and the tuber groups (*p* > 0.05).

### SyMRI results

Automated whole-brain segmentation showed that the Non-GM/WM/CSF (Non), Non/BPV%, and Non/ICV% were significantly higher, and MY/ICV% and MY/BPV% were significantly lower in the TSC patients (*p* < 0.05) than HCs, although patients with TSC tended to have lower BPV, ICV, GM, and WM volumes compared to HCs (Table 2).

**Table 2:** Comparison of SyMRI parameters of TSC patients and healthy controls

Variables	Healthy controls		Tuberous sclerosis		*p*-values
	Median	Min–max	Median	Min–max	
BPV (ml)	1278	1125–1502	1202	1033–1476	.860
ICV (ml)	1400	1235–1599	1399	1176–1629	.705
WM (ml)	378	301–550	398	343–538	.561
GM (ml)	855	707–1012	821	727–996	.820
CSF (ml)	109	75–309	91	66–212	.561
MY (ml)	109	81–138	93	85–113	.118
Non-WM/GM/CSF (ml)	15	7.7–65	20	15–43	.035*
%Non/BPV	1.15	0.6–4.7	1.6	1.1–3.1	.049*
%MY/ICV	7.8	6.4–9.4	6.7	5.9–9	.05*
%MY/BPV	8.7	6.9–10	7.7	6.8–9.7	.035*
%Non/ICV	1.1	0.6–4.3	1.5	1–2.7	.050*
R NABP MyC	28.55	24.8–34.8	25.6	22–30	.035*
R NABP PD	66.9	62.8–69.4	68.9	66–71.2	.023*
L NABP MyCvol	0.18	0.10–0.20	0.16	0.14–0.19	.017*
L NABP MyC	28.5	24.6–32.3	26	23.5–29.9	.001*
L NABP PD	66.9	64–69	68.6	66–70	.001*
R Caudate MyC	7.2	4.5–8.7	5.35	2–7.3	.004*
R Caudate PD	81	79.8–82.7	82.2	80.8–86.7	.005*
L Caudate MyC	5.2	1–8.3	7.2	5.7–10	.004*
L Caudate PD	80.7	79.1–82	81.9	79.5–85.4	.023*
L Thalamus MyCvol	0.07	0.04–0.10	0.055	0.03–0.09	.009*
Pons MyCvol	0.09	0.07–0.12	0.07	0.06–0.09	.000*
Pons MYC	21.85	18–29	16.7	13.6–20.4	.000*
Pons PD	70.95	66.1–74	74.7	69.4–76.7	.002*

Units: MY (ml), R1 and R2 (s-1), BPV (ml), ICV (ml), WM (ml), GM (ml), PD (pu)

*Represents significance is < 0.05

On myelin-colored maps, the MYFvol and MyC values were significantly lower, and PD values were significantly higher in the caudate nucleus, putamen, pons, and both hemispheric NABP in TSC patients compared to HCs (*p* < 0.05, Table 2).

**Fig. 2:**
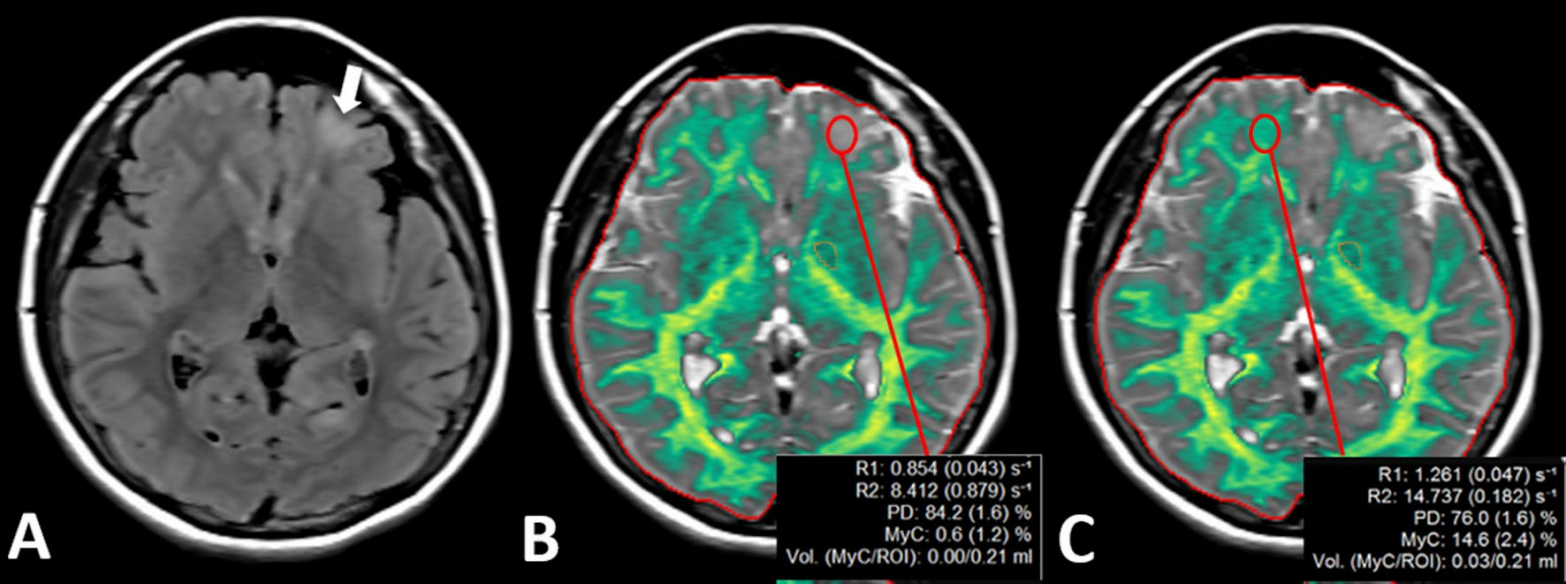
Images of 10-year-old patients with TSC. Subcortical tuber in the frontal lobe (**A**, white arrow) is seen on the axial FLAIR image. Myelin maps (**B** and **C**) show prominently decreased R1, R2, PD, MyC, and MyCvol values on the left side (**B**, red ROI) compared with the normal side (**C**, red ROI). Also, the myelin map does not assign the tuber as green like in other myelinated areas

In patients with TSC, the MYFvol, MyC, WMvol, WMFvol, R2, R1 values were significantly decreased in the subcortical part of the tubers compared to contralateral NABP, on myelin and WM quantification maps (*p* < 0.001, Additional file 1: Table S2, Fig. 2); however, GMvol, GMFvol, and PD values were significantly increased on the GM quantification map (p < 0.001, Additional file 1: Table S2).

**Fig. 3:**
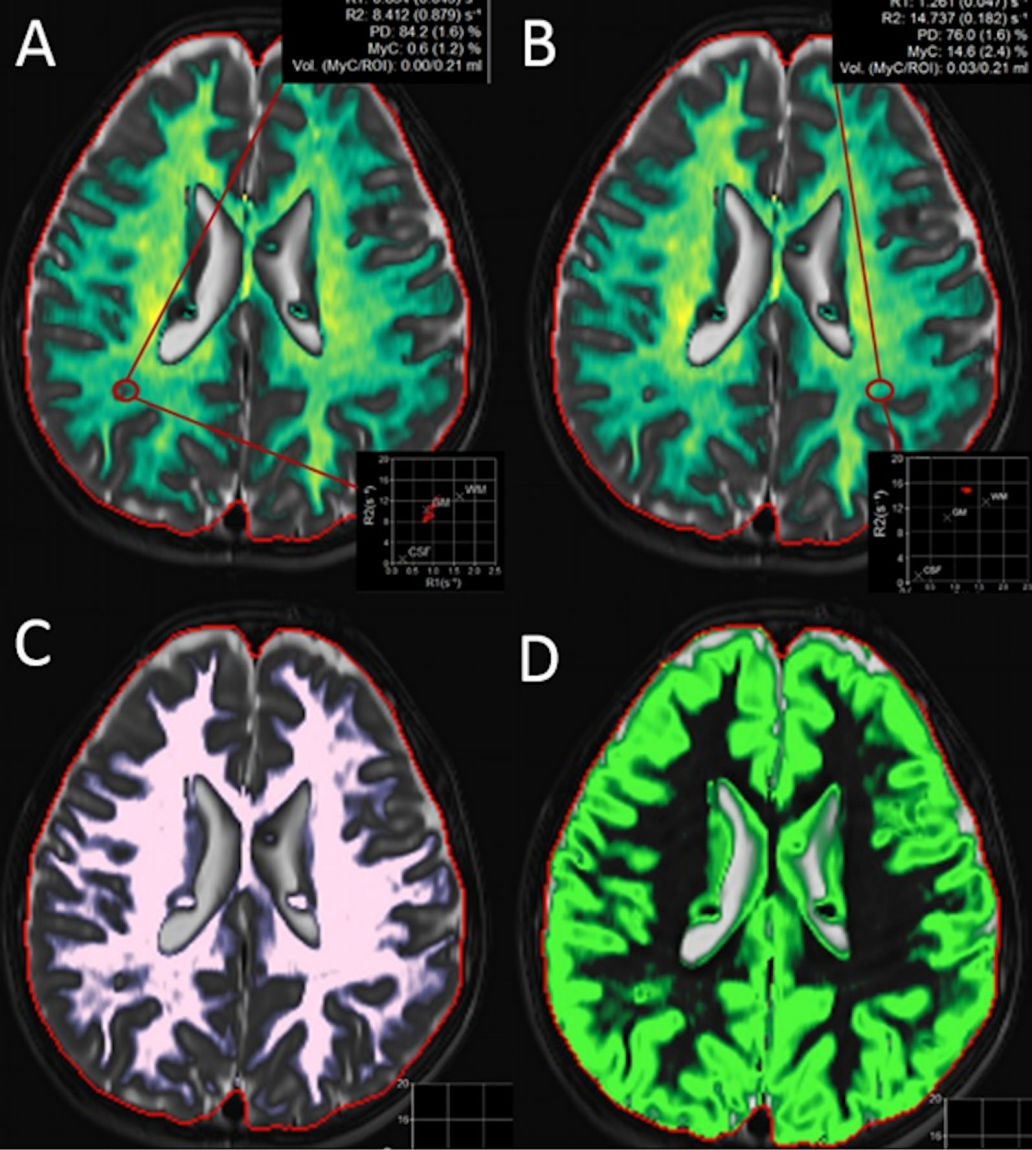
Myelin (**A**, **B**), WM (**C**), and GM (**D**) segmentation maps of a TSC patient. The subcortical part of the tuber on the right parietal white matter (**A**, red ROI) shows prominently decreased R1, R2, PD, MyC, and MyCvol values compared to the normal right side (**B**, red ROI). WM map (**C**) does not mark the subcortical tubers as WM. However, GM map (**D**) marks the subcortical tubers as GM

The observers noticed that cortical/subcortical tubers were automatically labeled on the Non-GM/WM/CSF, and GM quantification maps, but not labeled and calculated on WM and myelin quantification maps (Fig. 3 & Additional file 1: Fig S2). Also, the centrum of the subependymal nodules was automatically labeled as WM and the peripheral part of the subependymal nodules was labeled as myelin and GM on the corresponding quantification maps (Fig. 3 & Additional file 1: Fig. S2).

## Discussion

This study reports the first qualitative and quantitative imaging analysis of TSC patients with SyMRI. We found significantly higher Non-GM/WM/CSF (Non), Non/BPV%, Non/ICV%, and lower MY/ICV%, MY/BPV% values, and alterations in myelin. Patients with TSC tended to have lower BPV, ICV, GM, and WM than HCs in our study, similar to previously reported by Riddler et al. who demonstrated significant GM alterations in basal ganglia, thalamus, and decreased WM volumes in TSC patients using computational morphometry by automated anatomical labeling [17]. By our exclusion criteria, we avoided the possible incremental effects of SEGA in this study. Therefore, our morphometric findings may more accurately reflect morphological abnormalities by the disease itself in TSC.

Diffusion tensor imaging (DTI) is another technique that can demonstrate microstructural tissue changes in certain diseases [18, 19]. Elevated apparent diffusion coefficient (ADC) and reduced fractional anisotropy (FA) throughout the WM tracts suggested alterations in the integrity of WM in TSC. Regarding the NABP in TSC, some studies showed the higher mean diffusivity (MD) and the lower FA values in the supratentorial NABP and perituberal WM in patients with TSC [20, 21]. Our results were similar to these DTI findings, showing that all values except PD were significantly decreased in the subcortical part of tubers and NABP on myelin quantification maps using SyMRI, in TSC patients. These findings may point to underlying myelin and/or axonal structural changes, similar to the studies conducted in MS, and neurofibromatosis type-1 patients [13, 22, 23], and also may explain the global neurocognitive deficits in TSC patients [6].

Histopathological studies reported increased heterotopic and dysplastic neurons with extensive astrocyte pathology in tubers and perituberal tissue [24, 25]. A study of histopathological correlation of TSC with DTI study showed that reduced FA and increased MD in the perituberal WM corresponded to reduced myelin in dysplastic tissue [26] alike to the previous rodent models [27, 28]. Validation of myelin values obtained by SyMRI was proved by good correlations with histological specimens [29] and other myelin measurement methods in healthy subjects [30]. Also, measurement of myelin with SyMRI was supported with Magnetization Transfer Saturation Index and with postmortem brain studies [30]. A prominent reduction in myelin content of NABP in TSC has also been shown herein, although the effects of astrocytosis or neuronal ectopia are not known because of lacking histopathological correlation. Correlative investigations of histological changes in myelin with SyMRI may shed light on actual histopathological contributions to these quantitative changes. Moreover, given that use of mTORC inhibitors changed the DTI metrics [31, 32] in WM, there is also a possible role for SyMRI in the follow-up of response to treatment in these patients.

Patients with TSC are likely to develop SEGA in up to 20% of TSC patients. Patients who have a SEGA may initially have no signs or symptoms of having a brain tumor, but only have symptoms when the tumor grows large enough to obstruct the CSF flow. In regard to SEGA, these children should undergo regular follow-up brain MRIs until approximately the age of 25 years after when the intervals can be extended under stable conditions [5]. Brain MRI every 1–3 years was also recommended by the 2012 International TSC Consensus Conference [5]. However, long acquisition times pose a challenge in imaging these patients due to accompanying cognitive and neuropsychiatric disorders. With SyMRI, shorter scan times are possible after the baseline cMRI examination, as we already pointed out in our previous work on NF-1 [13]. Automatically segmented volume calculations of the brain and quantitative data obtained with SyMRI could also be very promising and useful in follow-up comparisons. The obtained Non-GM/WM/CSF volume and percentage data may specifically provide a quantitative comparison of a load of subependymal nodules and cortical/subcortical tubers in patients' follow-up.

We performed this study using 2D Synthetic MRI due to the timing of this study; however, with recent developments a 3D imaging would give additional quantitative information such as regional cortical thickness and subcortical volumes, which showed good agreement with 3D synthetic T1-weighted images showed good agreement in conventional T1-weighted images [33, 34].

Several limitations exist in our study. First, this was a single-center feasibility study with relatively small sample size. This comes from the inclusion criteria of the patient group. We included only pediatric patients who did not require sedation in the MRI unit. Second, because the use of SyMRI was performed in a limited number of pediatric patients, we could not evaluate possible age effects on brain morphometry. We did not compare the SyMRI and conventional techniques for T1, T2 mapping because our protocol was limited to conventional sequences we used in the routine clinical practice. Neither correlations of volume measures with neuropsychological performance nor quantitative findings with histopathological abnormalities are available in this study due to pandemic situations that limited medical or surgical interventions.

## Conclusion

SyMRI provides quantitative information in patients with TSC reflecting changes in morphometry and tissue properties. Thus, SyMRI offers a promising tool by quantification of brain tissue and revealing multi-contrast sequences in a single acquisition with a reasonable scan time, especially in young children.

## Supplementary Information

**Additional file 1. Supplementary Table 1**. Demographic features of TSC patients and number of cortical/subcortical tubers according to localization on cMRI.

## Data Availability

The data are included in this publication.

## Abbreviations

AT: Acquisition time
BG: Basal ganglia
BPV: Brain parenchymal volume
cMRI: Conventional MRI
CSF: Cerebrospinal fluid
FDA: Food and Drug Administration
GM: Gray matter
GMFvol: The mean amount of GM within a single voxel
GP: Globus pallidum
HC: Healthy control
ICV: Intracranial volume
mTORC: Mammalian target of rapamycin complex
MY: Myelin
MyC: Myelin-correlated compound volume
MyCvol: Myelin-correlated fraction volume
MYF: Myelin fraction (MY/BPV)
NABP: Normal-appearing brain parenchyma
PD: Proton density
R1: Longitudinal relaxation rate
R2: Transverse relaxation rate
SEGA: Subependymal giant cell astrocytomas
Sy: Synthetic
SyMRI: Synthetic MRI
TSC: Tuberous sclerosis complex
WM: White matter
WMFvol: The mean amount of WM within a single voxel
