# Supplementary material for: Synthetic MRI in children with tuberous sclerosis complex
Source: Insights Imaging. 2022 Jul 7;13:115. doi: 10.1186/s13244-022-01219-2 (PMC9263007; doi:10.1186/s13244-022-01219-2)
Supplement: Supplementary file 1 — Additional file 1. Supplementary Table 1. Demographic features of TSC patients and number of cortical/subcortical tubers according to localization on cMRI. [file 13244_2022_1219_MOESM1_ESM.docx]

**Supplementary Table 1.** Demographic features of TSC patients and number of cortical/subcortical tubers according to localization on cMRI.

| TSC patients | Gender | Age | Number of tubers | Group of tubers | Frontal | Temporal | Parietal | Occipital |
| --- | --- | --- | --- | --- | --- | --- | --- | --- |
| 1 | F | 9 | 13 | 2 | 7 | 2 | 4 | 0 |
| 2 | F | 14 | 15 | 2 | 9 | 1 | 5 | 0 |
| 3 | M | 14 | 32 | 3 | 16 | 6 | 8 | 2 |
| 4 | F | 11 | 28 | 3 | 10 | 7 | 7 | 4 |
| 5 | M | 12 | 45 | 3 | 19 | 9 | 9 | 8 |
| 6 | F | 7 | 3 | 1 | 1 | 0 | 2 | 0 |
| 7 | F | 13 | 5 | 1 | 4 | 0 | 1 | 0 |
| 8 | F | 11 | 11 | 2 | 6 | 4 | 1 | 0 |
| 9 | M | 17 | 42 | 3 | 20 | 6 | 11 | 5 |
| 10 | M | 7 | 37 | 3 | 22 | 6 | 4 | 5 |
